# Sustainable Eggshell-Based Amorphous Calcium Phosphate Scaffolds and Membrane Protein Hydrogel for Regeneration in Rabbit Femoral and Calvarial Defects

**DOI:** 10.1021/acsami.5c25453

**Published:** 2026-03-05

**Authors:** Qianli Ma, Katrin Anja Staudigel, Kristaps Rubenis, Zhaoyue Fu, Torben Hildebrand, Markuss Zaldenieks, Ólafur Eysteinn Sigurjónsson, Liebert Parreiras Nogueira, Janis Locs, Dagnija Loca, Lihua Chen, Håvard Jostein Haugen

**Affiliations:** † Department of Biomaterials, Faculty of Dentistry, 6305 University of Oslo, Oslo 0317, Norway; ‡ Department of Microphysiological Systems, Institute of Biomedical Engineering, 9188 Faculty of Medicine of the Eberhard Karls University of Tübingen, Waldhörnlestr. 22, 72072 Tübingen, Germany; § Institute of Biomaterials and Bioengineering, Faculty of Natural Sciences and Technology, 468614 Riga Technical University, Paula Valdena Street 3 k-1, Riga LV-1048, Latvia; ∥ Baltic Biomaterials Centre of Excellence, Headquarters at Riga Technical University, Riga LV-1048, Latvia; ⊥ Department of Immunology, School of Basic Medicine, 12644 Air Force Medical University, Xi’an 710032, P. R. China; # School of Science and Engineering, Reykjavík University, Reykjavík 102, Iceland; ∇ The Blood Bank, Landspitali-The National University Hospital of Iceland, Reykjavík 105, Iceland; ○ Oral Research Laboratory, Faculty of Dentistry, University of Oslo, Oslo 0317, Norway

**Keywords:** amorphous calcium phosphate, bone regeneration, critical-sized bone defect, eggshell, porous scaffold

## Abstract

Critical-sized bone defects require grafts with ideal spatial supporting. We upcycled eggshell waste sustainably into porous amorphous calcium phosphate (ACP) scaffolds and evaluated their early bone-regeneration performance alone and in combination with flexible and bioactive adjuncts *in vivo*. ACP scaffolds with ∼60% porosity were fabricated by pressure-densification, using ice microspheres as space holders. Before implantation, ACP scaffolds were vacuum-infiltrated with a hyaluronic acid (HA) hydrogel to minimize internal voids. Bilateral critical-size femoral and calvarial defects were created in New Zealand White rabbits and reconstructed with one of four treatments: (1) Sham, (2) ACP scaffold (ACP), (3) ACP infused with HA hydrogel containing a proline-rich peptide P2 (ACP + HA/P2), or (4) ACP infused with HA/P2 supplemented with soluble eggshell membrane proteins (prepared by our lab, ACP + HA/P2 + SEPs). After 6 weeks, micro-CT and histology were performed. All ACP-containing groups showed greater defect filling and mineralization than the Sham group. Micro-CT analysis demonstrated that ACP + HA/P2 + SEPs achieved the highest bone volume and trabecular number and the lowest trabecular separation. Histology confirmed more extensive mineralized collagenous deposition on ACP scaffolds and a more integrated bone–material fusion zone in this group. In femoral defects with graft displacement, ACP scaffolds induced cortical–scaffold bone bridging within the marrow, most evident with SEPs. Eggshell-derived ACP scaffolds, particularly when paired with SEP-enriched HA hydrogel, enhance early regeneration of critical-sized bone defects. Upcycling the inorganic and organic constituents of eggshell waste yields a complementary, single-origin biomaterials package for sustainable bone repair.

## Graphical abstract

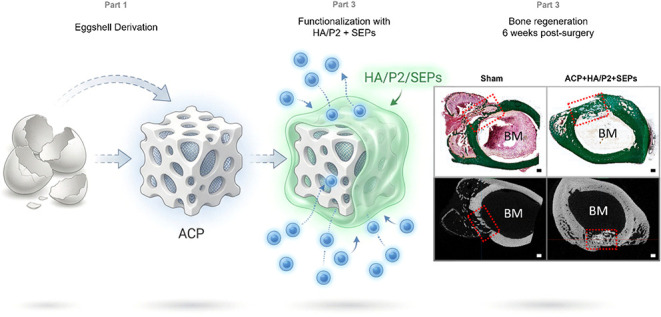

## 1 Introduction

Addressing critical-size bone defects remains a major challenge in regenerative medicine, orthopedics, and dental surgery. Conventional graft options, autograft, allograft, and xenograft, are constrained by donor-site morbidity, limited availability, infection risk, high biological and economic costs, environmental burden, and potential ethical issues,^1–4^ motivating a shift toward biomimetic synthetic materials.^5^ Given that *de novo* synthesis can involve harmful reagents, upstream pollution, and energy preconsumption, upcycling municipal solid food waste into biomimetic graft materials is an appealing strategy for sustainable biomaterials development.^6^ In this context, sustainable biomaterials aim to reduce environmental burden, inspiring researchers to contribute to eco-friendly solutions, while maintaining clinical performance, for example, by upcycling calcium-rich waste streams, such as eggshells into well-characterized calcium phosphate grafts,^6^ and by minimizing energy demand during fabrication through low-temperature processing routes that avoid high-temperature sintering yet still yield interconnected porous ceramics and preserve metastable, potentially bioactive phases.

Eggshells are a high-volume (millions of tons annually) biomineralized food waste primarily composed of CaCO_3_ (94%) with minor Ca_3_(PO_4_)_2_ (1%) and MgCO_3_ (1%), and contain ppm-level Mg, Sr, and S,^7–11^ making them an ideal precursor for trace element-containing calcium phosphate (CaP) product. We previously converted eggshells into amorphous calcium phosphate (ACP) using alkaline-acid chemistry, yielding nanostructured clusters (average diameter 13.23 ± 9.66 μm) with a high specific surface area (159.6 m^2^/g). Importantly, this synthesis route effectively retained Mg (2.20 ± 0.22 g/kg) and Sr (236 ± 24 mg/kg) at levels close to those in natural bone.^12,13^ These essential trace elements integrated into the amorphous matrix also contributed to the resistance of ACP recrystallization.^14^ Moreover, the amorphous structure of ACP supports controlled release of Ca/P ions and extracellular matrix (ECM) mineralization, while the retained trace elements further promote vascularization and neo-osteogenesis.^12,15^ However, ACP particles in powder form have suboptimal handling properties and limited space-maintenance capacity. Constructing ACP scaffolds with an interconnected architecture can enhance surgical maneuverability and provide space for the in-growth of mineralized tissue. Conventional space holders typically require thermal or aqueous removal, risking ACP recrystallization.^16^ To address this issue, ice microspheres were introduced as space holders, densified at subzero temperatures, and removed by lyophilization, an approach designed to preserve ACP amorphousness by avoiding exposure to heat or prolonged contact with water.

Beyond the inorganic phase, the organic fraction of eggshell waste, soluble eggshell membrane proteins (SEPs), provides complementary bioactivity, enhancing proliferation and extracellular matrix mineralization in osteogenic lineages and is deliverable within a hyaluronic acid (HA) hydrogel.^17^ Constructing porous eggshell-derived ACP scaffolds infused with SEP-containing P2 peptide-functionalized HA hydrogel creates a single-origin inorganic–organic biomaterial package that integrates structural guidance with a supportive biological microenvironment. Beyond signaling, this hydrogel-integrated system provides essential biomaterial confinement within the defect site, acting as a physical seal to exclude fibrous tissue infiltration and ensuring the spatial stability of the loaded bioactive signals.^18,19^ Such confinement is a critical, yet often overlooked, confounder in guided bone regeneration (GBR) that dictates the efficiency of localized osteogenesis. While advanced *in vitro* systems yield valuable mechanistic insights, *in vivo* assessment is essential to capture the coupled effects of loading, perfusion, and host immune responses.^20^ The rabbit femoral defect provides a load-bearing, well-perfused model under physiologic cyclic loading during ambulation, whereas the calvarial defect offers a complementary nonload-bearing context.^21–23^

In this study, we fabricated porous eggshell-derived ACP scaffolds using an ice microsphere-templated densification process at subzero temperatures. Distinct from earlier methods in which ACP loses its amorphous phase or is embedded in hybrids,^24–26^ this work produces a pure, porous ACP scaffold that retains its amorphous character and intrinsic osteogenic activity. We assessed the regenerative performance of the developed scaffolds in rabbit critical-sized femoral and calvarial defects with and without an SEP-containing HA hydrogel. We hypothesized that the complementary inorganic (ACP) and organic (SEPs) components sourced from the same waste stream would synergistically enhance early bone regeneration and bone–graft integration while advancing a sustainable pathway for bone-graft materials. The rationale for using eggshell-derived ACP is its unique physicochemical advantages as a biomimetic mineralization precursor.^15,16^ Unlike its crystalline counterparts, the metastable amorphous state allows for a rapid dissolution–reprecipitation cascade upon implantation, creating a localized ionic supersaturation that facilitates the *in situ* formation of bone-like apatite.^27^ Furthermore, the multivalent trace elements (Mg and Sr) in Eggshell ACP are not merely structural components but function as bioactive ion signals that can modulate the host osteoimmunomodulatory environment and activate crucial osteogenic pathways, such as the BMP-2/Smad or Wnt/β-catenin signaling cascades.^28,29^ This orchestrated ion–cell interaction is hypothesized to synergistically promote the recruitment and lineage commitment of bone marrow mesenchymal stem cells (bMSCs) within a porous scaffold architecture (Chart 1).

**Chart 1:**
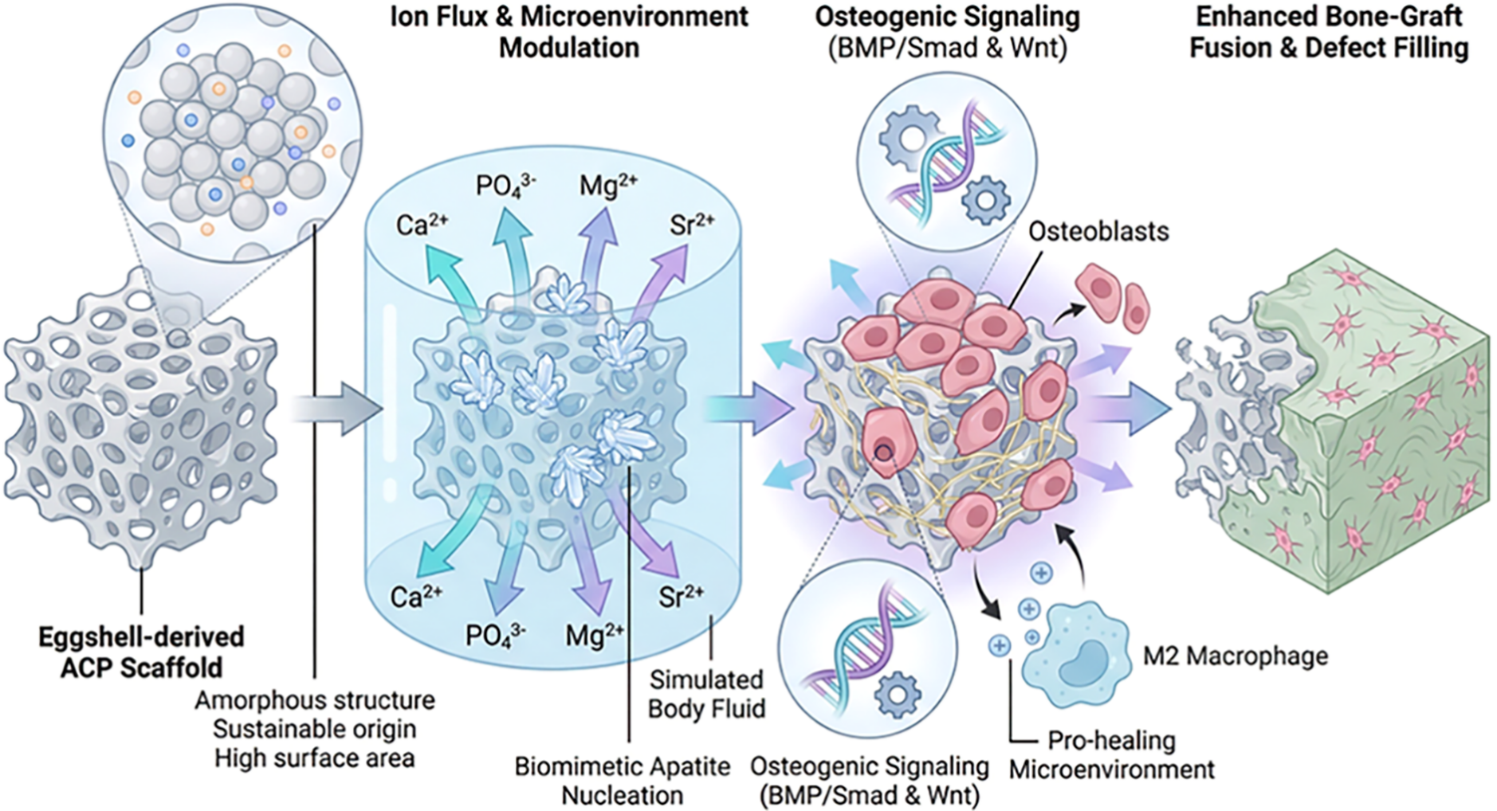
Diagram of ACP-Promoted Biomineralization and Bone-Material Fusion[Fn c1fn1]

a Originally created by authors on the Artlist platform with full license.

## 2 Materials and Methods

### 2.1 Ethics Approval and Consent to Participate

All animal experiments were performed in accordance with ARRIVE guidelines 2.0 and the Guide for the Care and Use of Laboratory Animals published by the National Institutes of Health (NIH). The study protocol was reviewed and approved by the Animal Experiment Ethics Committee of the Air Force Medical University (Formerly known as Fourth Military Medical University, Approval No. KY20194055–2023 kq-057). All animals were cared for and supervised in strict accordance with the Guide for the Care and Use of Laboratory Animals of Air Force Medical University (2018). Every effort was made to minimize animal suffering and limit the number of animals used. Consent to participate is not applicable.

### 2.2 Synthesis of Amorphous Calcium Phosphate

The synthesis of Eggshell ACP powder was reported in our previous work.^14,30^ In brief, eggshells “Balticovo” (Iecava, Latvia) were thermally treated at 900 °C for 1 h to remove organic components and were transformed into CaO_3_. Then, 5.55 g of CaO was added to 600 mL of deionized water (dH_2_O), and the mixture was stirred continuously at 200 rpm to form a white suspension. After 10 min (min), 12.47 mL of H_3_PO_4_ solution (4.76 M) was added to the suspension. After 10 min, the stirring speed was increased to 600 rpm, and 91.5 mL of NaOH aqueous solution (2 M) was rapidly added to allow the formation of a white precipitate with the rapid increase of pH value to ∼11.0. After 5 min, the precipitate was filtered and washed with deionized water (dH_2_O). Then, the precipitate was frozen in liquid nitrogen and afterward freeze-dried in a β 2–8 LSCplus freeze-dryer (Martin Christ Gefriertrocknungsanlagen GmbH, Osterode am Harz, Germany) for 72 h.

### 2.3 Fabrication of Porous ACP Scaffold

To prepare porous ACP ceramic scaffolds, ice microspheres with a diameter between 200 and 400 μm were first prepared by spraying water into a liquid-nitrogen bath (polystyrene container) containing stacked sieves (400 μm over 200 μm). After the sieve was sprayed, the 400 μm sieve was removed, and particles retained on the 200 μm sieve were collected and mixed with ACP powder. The following eq 1 was used to calculate the amount of ice microspheres required to produce ACP scaffolds with specific porosity

1 $$\phi =\frac{(m_{spaceholder}/\rho_{spaceholder})}{\left(m_{spaceholder}/\rho_{spaceholder}+m_{material}/\rho_{material}\right)}$$

ϕ is the porosity of the material in volume %, *m*_space holder_ is the mass of the space holder, ρ_space holder_ is the density of the space holder, *m*_material_ is the mass of the material, and ρ_material_ is the density of the material. To produce 60% porous ACP ceramic scaffolds, the calculated amounts of ice microspheres and ACP powder, precooled to approximately – 50 °C, were mixed in a cooled polypropylene container. The total mass of the ACP and ice sphere mixture was 0.069 g for a 5 mm diameter die. This mixture was then transferred to a precooled pressing die at – 50 °C, which was transferred to a PW 100 ES two-column electrohydraulic laboratory press (P/O/WEBER, Remshalden, Germany), where a uniaxial pressure of 1500 MPa was applied to the mixture for 1 min. After compaction, the pressure on the samples was slowly released and the scaffolds were allowed to warm to room temperature on tissue paper, with periodic repositioning and rotation to facilitate moisture removal. Prior to in vivo implantation, ACP porous scaffolds were sterilized by E-beam irradiation at 25 kGy (performed by Yangling Hesheng Nuclear Radiation Technology Co., Ltd., Xi’an, China). The characterization of ACP scaffolds was performed using micro-CT, scanning electron microscopy (SEM), and X-ray diffraction (XRD, Malvern Panalytical Aeris, Malvern Panalytical, United Kingdom).

### 2.4 Extraction of SEPs

Eggshell membrane (ESM) was collected from freshly discarded eggshells in the kitchen, and the SEPs were extracted as previously reported.^17^ Briefly, the ESM was cut into small pieces and added to a 1.25 M aqueous solution of 3-mercaptopropionic acid (3-MPA) in a 10% (v/v) aqueous acetic acid solution. After pH adjustment (pH adjusted to 5.0 and then to 7 to precipitate proteins) and precipitation washing, SEPs were dissolved in distilled water and filtered through 0.22 μm sterile syringe filters (CLS431224, Corning). Subsequently, the SEP solution was lyophilized and stored at −20 °C. The freeze-dried SEPs powder was dissolved in sterile dH_2_O instantly before use.

### 2.5 Hyaluronic Acid Hydrogel with P2 Peptide Preparation

HA hydrogel was synthesized as previously reported.^31^ In brief, high-molecular-weight hyaluronic acid (HA) (MW = 1.5 MDa, IV = 22.2 m^3^/kg, SyrHA, Geneva, Switzerland) was dissolved at 10 w/v% in 0.3 M NaOH under manual agitation. 1.6 v/v% 1,4-butanediol diglycidyl ether (BDDE, Merck KGaA, Germany) was added and stirred in. The solution was incubated for 4 h at 40 °C in a closed container. Subsequently, the hydrogel was dialyzed and then granulated by extrusion through a 130 μm mesh. After that, the HA hydrogel was diluted with distilled water with Proline-rich P2 peptide (sequence PLV PSQ PLV PSQ PLV PSQ PQ PPLPP, synthesized by Pepmic Co., Ltd., Jiangsu, China, covered by the patent^32^) to reach a final concentration of 20 mg/mL (HA) and 50 mg/mL (P2), respectively. All manufacturing steps were conducted under Good Laboratory Practice (GLP) conditions in an ISO 13485:2016 certified facility. HA/P2 Samples were filled into syringes and autoclaved at 121 °C for 15 min before biological application.

### 2.6 Graft Material Preparation

Four different graft settings were applied for the bone defects *in vivo*: (1) Sham; (2) ACP scaffold; (3) ACP+HA/P2; and (4) ACP+HA/P2+SEPs. To prepare the HA/P2+SEPs hydrogel, the HA/P2 hydrogel was mixed with the SEPs solution to achieve a final SEPs concentration of 50 μg/mL. For Grafts (3) and (4), ACP scaffolds were placed in a syringe filled with HA/P2 hydrogel (with or without SEPs). A vacuum was then applied to this system to expel air from the ACP scaffolds and facilitate the infiltration of the HA/P2 hydrogel into them. All of these procedures were conducted immediately prior to the in vivo *application to maintain* the amorphous characteristics of the ACP material.

### 2.7 Femoral and Calvarial Critical-Sized Defect Preparation in the Rabbit Model

Ten five-month-old New Zealand rabbits (specific-pathogen-free grade), weighing between 2.5 and 3.0 kg, were obtained from the Laboratory Animal Center of the Air Force Medical University. All rabbits were administered isoflurane with a facemask as general anesthesia and lidocaine for local anesthesia (Sigma–Aldrich). Surgical steps are explicit in the flowchart of this study (Figure 1). (1) To expose the femurs (6 rabbits included), a 4.0 cm long-axis incision through the skin and muscles was made, followed by periosteum separation. Two critical-sized bone defects (Ø5.0 mm) were made on the distal femur on each side (4 defects on femurs per rabbit) using a W&H planter (W&H Dentalwerk, Austria) and trephine bur (Ø5.0 mm, Neodent, Straumann group, Brazil) under cold (4 °C) normal saline cooling. The full-thickness bone chips in the defect area were removed and different graft materials were placed in the defects (Sham, ACP scaffold, ACP scaffold + HA/P2, and ACP scaffold + HA/P2 + SEPs; the placement of materials switched clockwise for each rabbit). Bio-Gide (Geistlich, Switzerland) GBR membranes were cut to 3.0 × 1.5 cm and placed over the bone defects, with grafts placed. (2) To expose the top cranium plate (4 rabbits included), a 5.0 cm midsagittal incision through the skin and muscles was made, followed by Periosteum separation while avoiding any damage to the orbital area and dura mater. Three critical-sized bone defects (Ø5.0 mm) on each side were made on the cranium, and graft materials were placed in the defects (ACP scaffold, ACP scaffold + HA/P2, and ACP scaffold + HA/P2 + SEPs; the placement of materials switched clockwise for each side). Bio-Gide GBR membranes were cut into a 3.5 × 2.5 cm size and were covered on the bone defects with a graft placed. After wound closure, isoflurane inhalation was stopped and the animals were resuscitated. Cetylpyridine ointment (1.0 mg/g, Karo Pharma, Norway) was applied to the wound, and long-acting cefquinoxime suspension (0.5 g/20 mL, HuaChu Veterinary Pharm, China PR) was administered intramuscularly (2.5 mg/0.1 mL/kg) for 7 days (1 time daily) to prevent postoperative infection. Acetaminophen-treated water (1.0 mg/mL; Weifa, Norway) was administered to the rabbits as an analgesic strategy. After 6 weeks, all rabbits were euthanized under general anesthesia with intravenous administration of overdosed pentobarbital sodium (150 mg/kg body weight, Sigma–Aldrich). All femur and cranium samples were then obtained via the same surgical routes and fixed in 4% paraformaldehyde (PFA) for 72 h (femur) and 24 h (cranium), respectively. Subsequently, the samples were stored at 4 °C prior to analysis. During the postoperative feeding period (at day 41 and day 42), unilateral femur fractures developed in two rabbits. Samples affected by the fracture sites were excluded from subsequent analysis. Samples with obvious inflammation or graft displacement were excluded from statistical analysis.

**Figure 1:**
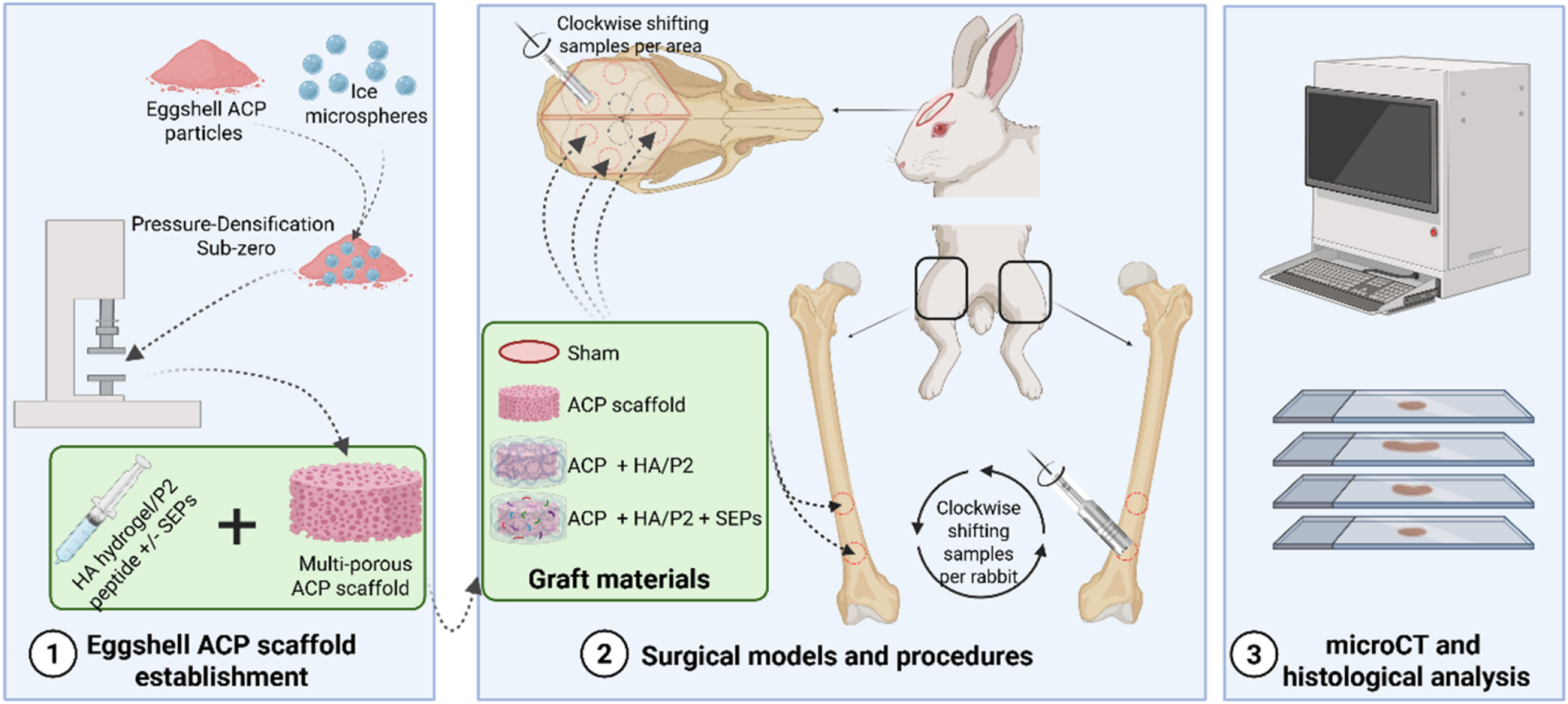
Flowchart of research design in materials preparation, surgical operations, and data analysis. P2, cross-linked hyaluronic acid hydrogel with P2 peptide; SEPs, soluble eggshell membrane proteins. Originally created by authors on the BioRender platform with full license

### 2.8 Micro-CT Analysis

(1) For ACP samples: ACP scaffolds (*n* = 4) were scanned using a micro-CT system (Skyscan 1172, Bruker Belgium SA, Kontich, Belgium) at 70 kV and 120 μA, with a rotation step of 0.31° and a 0.5 mm Al filter, yielding a final voxel size of 5.92 μm. Image reconstruction was performed in NRecon (v1.7.4.6) using the fast hierarchical back-projection (FHBP) algorithm with a 40% beam hardening correction and a ring artifact correction of 7. Reconstructed 8-bit data sets were processed in Fiji (ImageJ, NIH) using a nonlocal means filter (σ = 2, smoothing factor = 3) followed by an unsharp mask (radius = 1.0 pixels, weight = 0.6). Scaffold visualization was performed in Dragonfly (v2024.1, Object Research Systems, Montréal, Canada), and quantified in CTAn (v1.23.0.2) after global thresholding based on Otsu’s method.
(2) For fixed rabbit samples: samples were subjected to the same micro-CT scanning system at 74 kV, 124 μA with a 0.5 mm Al filter, yielding a final voxel size of 10.5 μm. Scans were reconstructed using NRecon software, with subsequent analysis via CTAn software in the cylindrical region of interest (ROI) with a diameter of 4.0 mm for bone regenerative indicators, including ratio of bone volume vs total volume (BV/TV), bone surface vs bone volume (BS/BV), bone surface vs total volume (BS/TV), trabecular thickness (Tb.Th), trabecular separation (Tb.Sp), and trabecular number (Tb.N).

### 2.9 Histological Staining and Observation

After micro-CT analysis, all samples were rinsed three times in dH_2_O (1 h per round), dehydrated in ethanol with different concentrations (70, 70, 95, 95, 100, 100%, 24 h per round), and subsequently embedded in methyl-methacrylate resin (Technovit 7200 VLC, Exakt, Germany). Using a cutting-grinding unit (EXAKT 300 and EXAKT 400 CS grinder, Advanced Technologies, Germany), blocks were cut and ground (sandpaper from P400, P800, P1200, P2500 to P4000) to a final thickness of approximately 40 μm. Sections were subjected to hematoxylin and eosin (HE) and Masson Goldner Trichrome (MGT) staining. All stained histology slides were scanned using an AxioScan Z1 (Carl Zeiss, Germany) and were analyzed with Zen3 software (Carl Zeiss, Germany).

### 2.10 Statistics

All data obtained were plotted and analyzed using Prism 10.4.0 software (GraphPad Software). Quantitative variables assessed included BV/TV, BS/TV, Tb.Th, Tb.Sp, and Tb.N from micro-CT. The normality of the data distribution was evaluated by using the Shapiro-Wilk test. Final analyzed sample size per group: *n* = 4–5 for femurs and *n* = 4 for craniums after excluding individual samples that exhibited either obvious tissue inflammation or significant graft displacement, as these factors would confound the assessment of material-induced bone regeneration. Given the inherently small sample sizes typical of in vivo rabbit studies, which limit the statistical power of normality tests, we applied a strict decision framework. For normally distributed data, one-way analysis of variance (ANOVA) followed by Tukey’s multiple comparisons test was performed. For data that did not pass the normality test, the Kruskal–Wallis test followed by Dunn’s multiple comparison test was employed. Data from ACP characterization are expressed as the mean ± the standard deviation (SD) for continuous variables. Data from the micro-CT analysis are depicted in a box plot, with the median and 10th/90th percentiles indicated. Statistical significance was defined as *p* < 0.05. Detailed levels of significance and sample size per group were also provided in figure legends.

## 3 Results

### 3.1 Characterization of Porous ACP Scaffolds

Representative SEM and micro-CT images of the ACP scaffolds are shown in Figure 2. After pressure-densification, the ACP scaffolds exhibited relatively flat top and bottom surfaces with fewer openings (Figure 2A). In micro-CT images, ACP scaffolds exhibited a layered structure with uniform pores (Figure 2B). Furthermore, quantitative analysis of micro-CT data revealed a hierarchical pore architecture, as indicated by pore- and throat-size distributions (Figure 2C). While small throats made pores fully accessible, the fraction of accessible pores decreased progressively with increasing throat size. A sharp transition was observed between 80 and 150 μm; beyond this threshold, most pores were inaccessible. With a throat size of *T*_50_ ≈ 114.6 μm, the fraction of accessible pores and nonaccessible pores is equal. This behavior was further reflected in the accessible volume of the scaffold (Figure 2D), which decayed sigmoidally and normalized to the total scaffold volume. The strut-thickness distribution showed a mean of approximately 71.18 ± 5.95 μm across the scaffolds (Table 1). Within individual scaffolds, the strut-thickness histogram exhibited a broader spread with an SD of 88.44 μm (Figure 2E).

**Figure 2:**
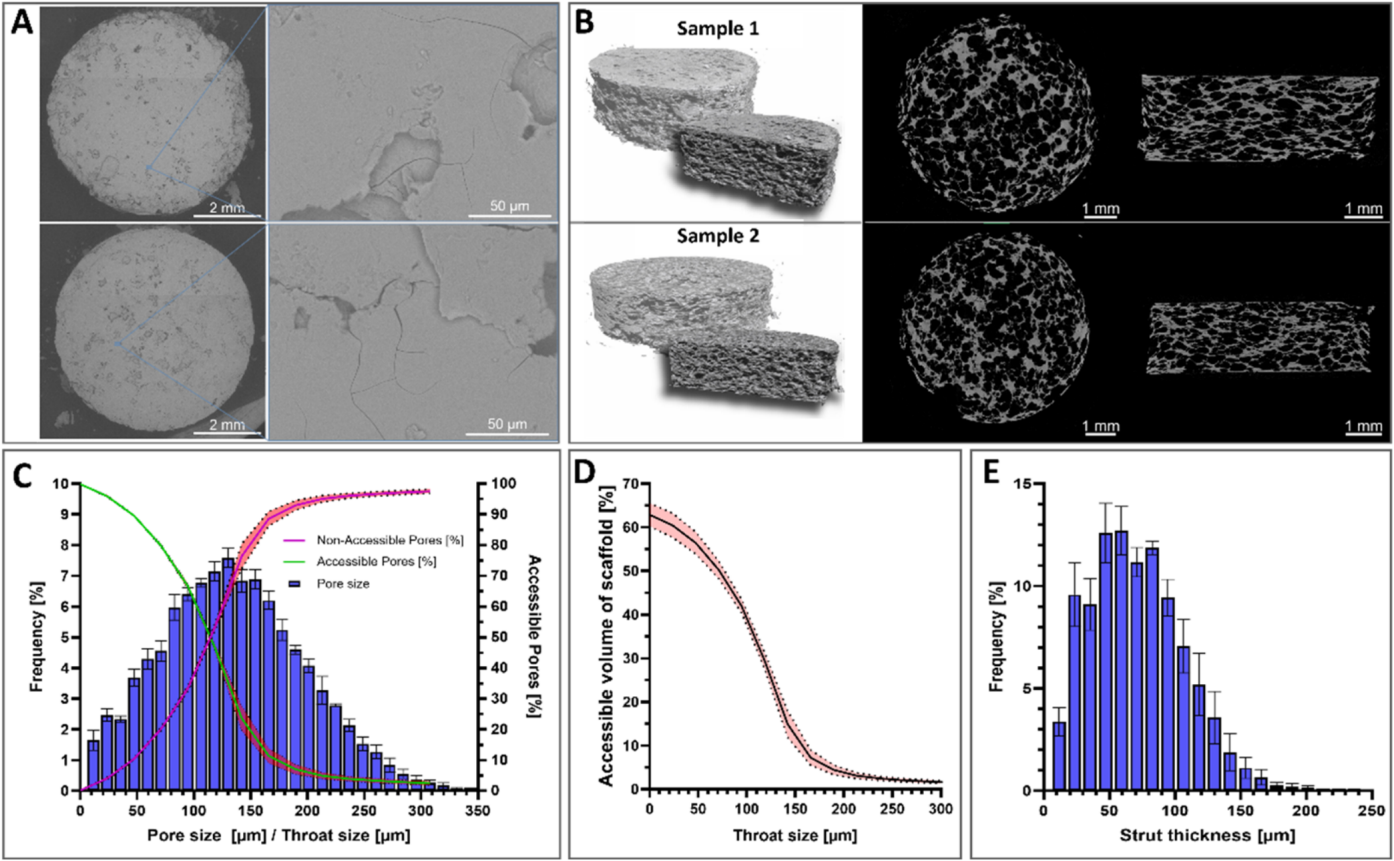
Micro-CT-based analysis of porosity of ACP scaffolds. (A) SEM images of the top and bottom surfaces of ACP scaffolds. (B) Reconstructive images of structures of ACP scaffolds. (C) Pore size distribution, normalized accessibility (green) and nonaccessibility (red) of pores with increasing throat size. (D) Total scaffold accessibility with increasing throat size. (E) Average strut-thickness distribution. *N* = 4.

**Table 1:** Morphological Analysis of Porous ACP Scaffolds

morphometrical parameters	mean ± SD^a^
object volume/total volume (Obj/TV)	37.05 ± 2.66%
struct thickness (St.Th)	71.18 ± 5.95 μm
pore size/struct separation (Po.Dm)	134.57 ± 1.51 μm
closed porosity (Po.V(cl))	0.16 ± 0.04%
open porosity (Po.V(op))	62.85 ± 2.32%
total porosity (Po.V(Tot))	62.95 ± 2.66%

a Standard deviation (SD) across all tested samples, not reflecting the distribution of indicators in one sample. *N* = 4.

According to our experimental design, using ice microspheres as a space holder, the prepared ACP scaffold is expected to have a porosity of approximately 60%. Micro-CT-based analysis showed that the spatial parameters of the prepared ACP scaffolds were very consistent, with a total porosity of 62.95 ± 2.66% and a pore size of 134.57 ± 1.51 μm across the scaffolds (Table 1). This porosity can be further characterized as being predominantly open, as shown in Figure 2D. These interconnected pores may permit the entry of blood and flexible graft materials, particularly structures smaller than 100 μm.

Given ACP’s sensitivity to water and heat generated by pressure-densification, XRD analysis was performed on the starting ACP powder and ACP scaffolds before and after E-beam sterilization. The XRD pattern (Figure 3) clearly showed that pressure-densification at subzero temperature and the use of ice microspheres as space holders facilitated the release of pressure-generated heat and limited the time that ACP was exposed to moisture, thereby protecting the amorphous phase of ACP scaffolds and potentially preserving related bioactivity.

**Figure 3:**
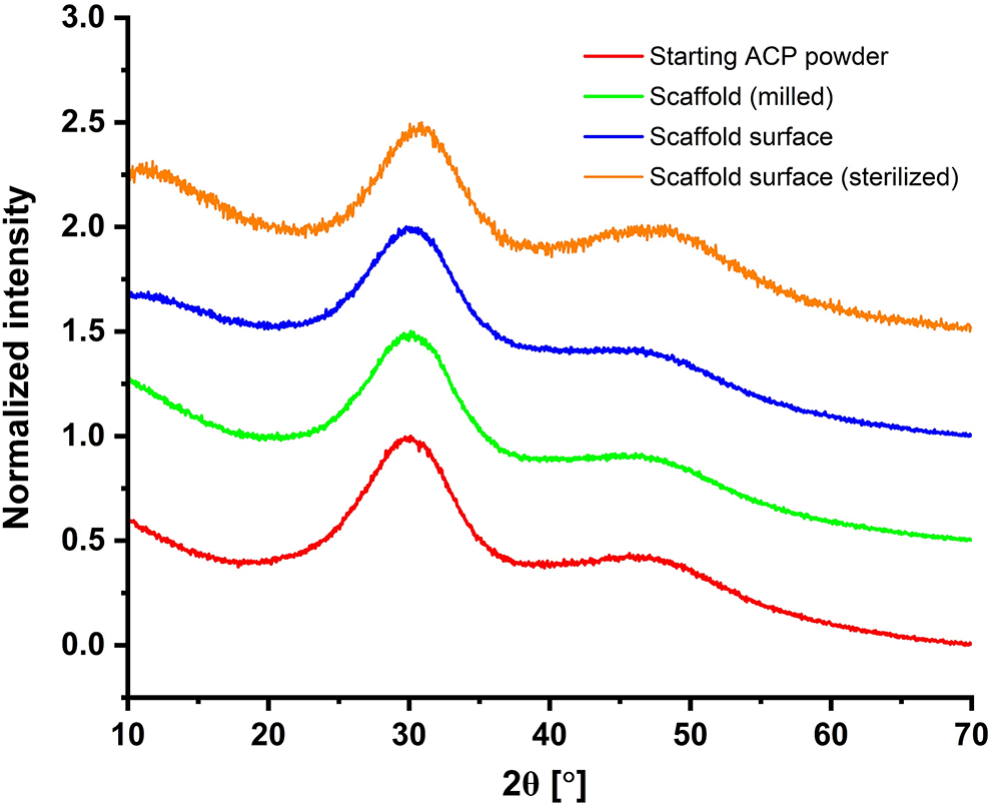
XRD patterns of the starting powder, the surface and bulk of the scaffold, as well as the scaffold surface after E-beam sterilization

### 3.2 Bone Regeneration of Critical-Sized Bone Defects in the Femoral Model

As shown in Figure 4 **A–D**, reconstructed micro-CT images showed minimal bone within Sham defects, whereas all ACP-included groups exhibited more bone formation. In longitudinal and cross-sectional views, only a thin, discontinuous bone layer was observed in Sham defects, whereas thicker bone-scaffold-integrated tissue could be observed in other groups (Figure 4E–L). Although preliminary qualitative micro-CT assessment did not reveal clear differences in bone formation among the ACP-containing groups, subsequent quantitative analysis demonstrated that the ACP + HA/P2 + SEPs group exhibited more favorable structural indices. To clarify bone regeneration across treatment groups, a cylindrical ROI with a 4 mm diameter perpendicular to the defect area was defined for quantitative analysis. By setting different intensity thresholds, all mineralized tissue and bone tissue without ACP scaffolds can be measured separately, as shown in Figure 5. All ACP-included groups showed a greater tendency toward bone regeneration (Figure 5A,F). At the same time, only the ACP and ACP + HA/P2 + SEPs treatments resulted in greater mineralized tissue formation than Sham (Figure 5A). Similarly, the combined application of ACP, HA/P2. SEPs also contributed to higher BS/TV and Tb.N as well as lower Tb.Sp, reflecting healthy bone structure with active remodeling (Figure 5C,E,F). After the ACP signals were excluded, the BV/TV of all ACP-included groups decreased. Although the trend in osteogenic indicators was similar across all groups, the statistical difference in BV/TV between Sham and ACP + HA/P2 + SEPs was no longer significant (Figure 5G). Intriguingly, the absence of the ACP signal did not hamper the superior BS/TV, Tb.Sp, and Tb.N of ACP + HA/P2 + SEPs than Sham (Figure 5I,K,L). It is also worth noting that the only combination of ACP and HA/P2 (in the absence of SEPs) resulted in a reduced tendency toward osteogenesis compared with ACP and ACP + HA/P2 + SEPs treatments (Figure 5A,C,F,I,L). All osteogenic indicators are summarized in Tables 2 and 3 for comparison.

**Figure 4:**
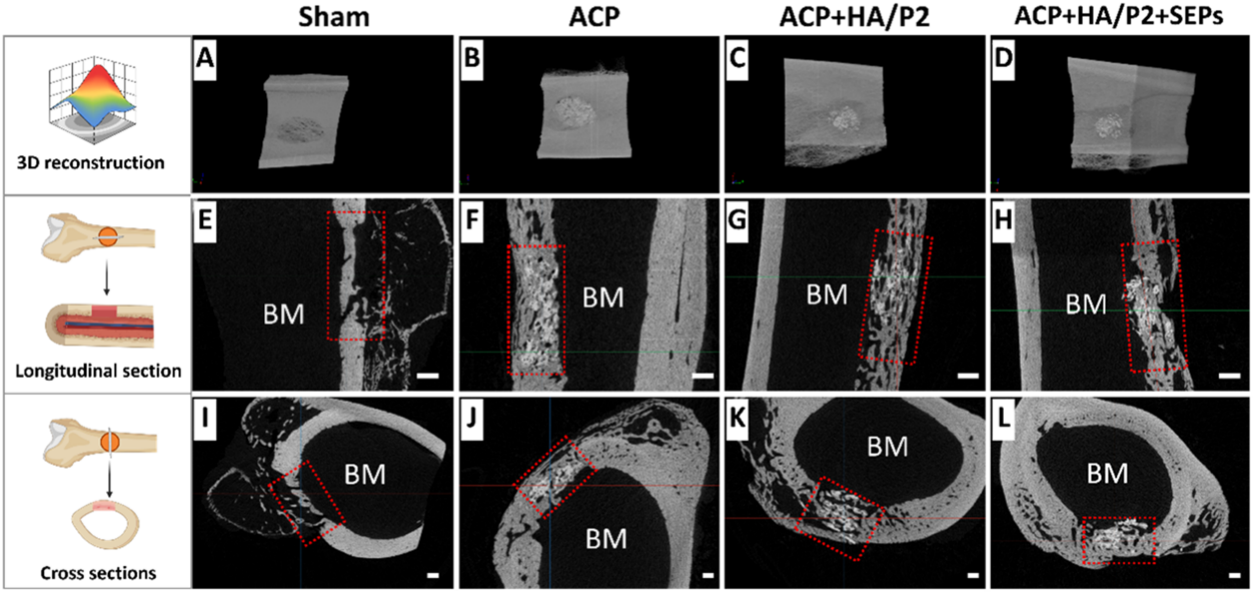
Micro-CT images and virtual sectioning of critical bone defects in rabbit femurs at 6 weeks postoperation. (A–D) Reconstructive images of bone regeneration in rabbit femur defects; (E–H) Bone regeneration and mineralization of longitudinal section of femur defects; (I–L) Bone regeneration and mineralization of cross-section of femur defects. Scale bar = 1.0 mm; Red dotted frame: original bone defect area.

**Figure 5:**
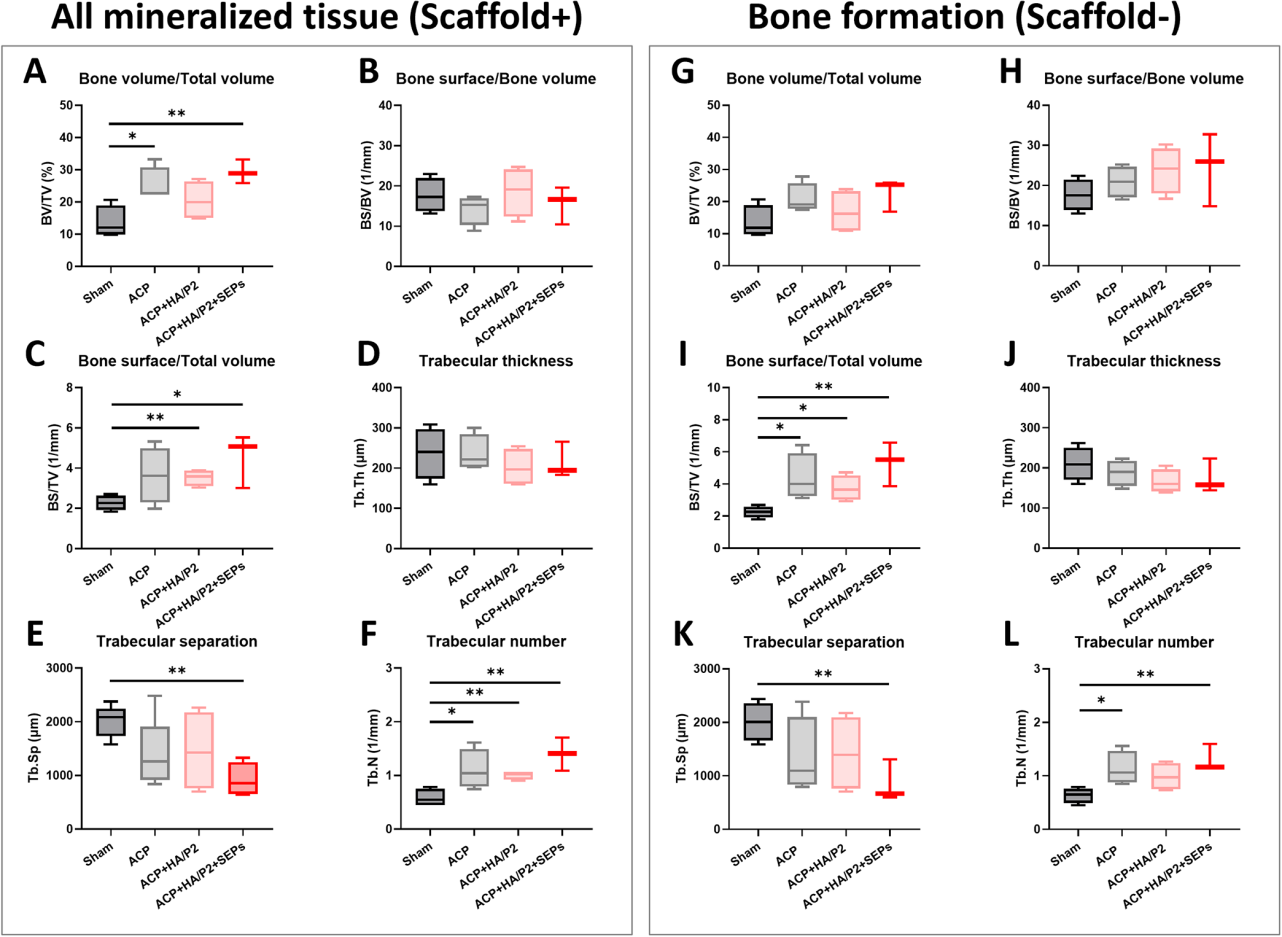
Micro-CT volumetric analysis of bone regeneration in a cylinder region of interest (ROI) with a diameter of 4.0 mm at 6 weeks postoperation in the rabbit femoral defect model. The bone formation was analyzed with (A, G) Bone volume/Total volume, (B, H) Bone surface/Bone volume, (C, I) Bone surface/Total volume, (D, J) Trabecular thickness, (E, K) Trabecular separation, and (F, L) Trabecular number. All analyses were performed on mineralized tissue with ACP scaffolds (in the left panel) and bone tissue without an ACP scaffold (in the right panel). *p < 0.05, **p < 0.01. *N*_sham_ = 5, *N*_ACP_ = 5, *N*_ACP+HA/P2_ = 4, and *N*_ACP+HA/P2+SEPs_ = 4.

**Table 2:** Quantitative Micro-CT Morphometric Parameters of Bone Regeneration at 6 Weeks Postoperation with the ACP Scaffold Signal

defect site	group	BV/TV (%)	BS/BV (mm^–1^)	BS/TV (mm^–1^)	Tb. Th (μm)	Tb. Sp (μm)	Tb.N (mm^–1^)
femoral defect	Sham	13.64 ± 4.34	17.65 ± 3.70	2.27 ± 0.32	237.04 ± 54.99	2009.96 ± 265.89	0.58 ± 0.14
	ACP	**25.24 ± 4.64** ^a^	14.19 ± 3.20	3.64 ± 1.21	236.38 ± 39.10	1382.32 ± 578.86	**1.11 ± 0.32** ^a^
	ACP+HA/P2	20.48 ± 5.33	18.53 ± 5.30	**3.52 ± 0.36** [Table-fn t2fn2]	201.90 ± 40.51	1451.69 ± 650.66	**1.00 ± 0.07** [Table-fn t2fn2]
	ACP+HA/P2+SEPs	**29.34 ± 3.00** [Table-fn t2fn2]	15.56 ± 3.81	4.54 ± 1.09^a^	214.64 ± 36.34	**919.47 ± 272.24** [Table-fn t2fn2]	**1.40 ± 0.25** [Table-fn t2fn2]
calvarial defect	ACP	22.55 ± 4.58	16.70 ± 1.32	3.73 ± 0.61	192.35 ± 9.47	895.75 ± 90.07	1.18 ± 0.25
	ACP+HA/P2	16.99 ± 4.10	19.41 ± 3.08	3.18 ± 0.28	174.36 ± 22.79	**1063.65 ± 54.81** ^c^	0.96 ± 0.12
	ACP+HA/P2+SEPs	22.46 ± 3.06	16.07 ± 2.30	3.55 ± 0.31	193.89 ± 20.86	1023.69 ± 47.21	1.16 ± 0.10

Femoral Defect:

a *p* < 0.05 vs Sham.

aa *p* < 0.01 vs Sham; Calvarial Defect:

c *p* < 0.05 vs ACP.

**Table 3:** Quantitative Micro-CT Morphometric Parameters of Bone Regeneration at 6 Weeks Postoperation without the ACP Scaffold Signal

defect site	group	BV/TV (%)	BS/BV (mm^–1^)	BS/TV (mm^–1^)	Tb. Th (μm)	Tb. Sp (μm)	Tb.N (mm^–1^)
femoral defect	Sham	13.50 ± 4.37	17.64 ± 3.39	2.25 ± 0.31	209.96 ± 36.08	2009.27 ± 309.82	0.64 ± 0.12
	ACP	20.85 ± 4.09	20.90 ± 3.42	**4.39 ± 1.25** ^a^	187.78 ± 27.80	1343.38 ± 621.85	**1.13 ± 0.27** ^a^
	ACP+HA/P2	16.82 ± 5.84	23.83 ± 5.01	**3.73 ± 0.68** ^a^	166.11 ± 25.18	1416.89 ± 612.11	0.99 ± 0.23
	ACP+HA/P2+SEPs	22.70 ± 4.15	24.52 ± 7.38	**5.31 ± 1.12** [Table-fn t3fn2]	175.35 ± 34.33	**857.78 ± 319.94** [Table-fn t3fn2]	**1.31 ± 0.20** [Table-fn t3fn2]
calvarial defect	ACP	9.38 ± 3.85	49.30 ± 10.67	4.24 ± 1.05	100.27 ± 18.84	845.51 ± 87.05	0.90 ± 0.26
	ACP+HA/P2	8.58 ± 1.08	38.25 ± 6.11	3.25 ± 0.52	119.17 ± 12.62	1085.87 ± 106.13^c^	0.72 ± 0.09
	ACP+HA/P2+SEPs	**12.02 ± 1.42** ^d^	35.56 ± 1.02	4.27 ± 0.42	129.61 ± 2.93	1044.76 ± 27.98	0.93 ± 0.13

Femoral Defect:

a *p* < 0.05 vs Sham.

aa *p* < 0.01 vs Sham; Calvarial Defect:

c *p* < 0.05 vs ACP.

d *p* < 0.05 vs ACP+HA/P2.

More details about the microstructures in the bone defect areas were provided by histological staining. As shown in HE staining (Figure 6, Upper panel), no apparent inflammatory infiltration was observed in any group, indicating that the inflammatory microenvironment did not significantly challenge the bone repair process. In Sham defects, only loose bone formation was observed with large cavities within the defects (Figure 6A,E,I). On the other hand, although all ACP-included groups showed more intact bone structures, the interactions between bone and materials differed across groups, which provided direct evidence of the physical confinement provided by the HA/P2 hydrogel. In the ACP-only group, new bone formation was largely restricted to discrete connections on the scaffold surface, with significant interstitial spaces occupied by marrow-like structures. In contrast, the ACP+HA/P2+SEPs group exhibited superior space-filling efficiency: the ACP scaffolds were almost entirely encapsulated and embedded within a thick, continuous layer of mature mineralized bone (green-stained in MGT staining; Figure 6T,X). Notably, no significant fibrous tissue infiltration was observed within the scaffold architecture in the hydrogel-treated groups, confirming the effective sealing and exclusionary function of the hydrogel-based confinement system.

**Figure 6:**
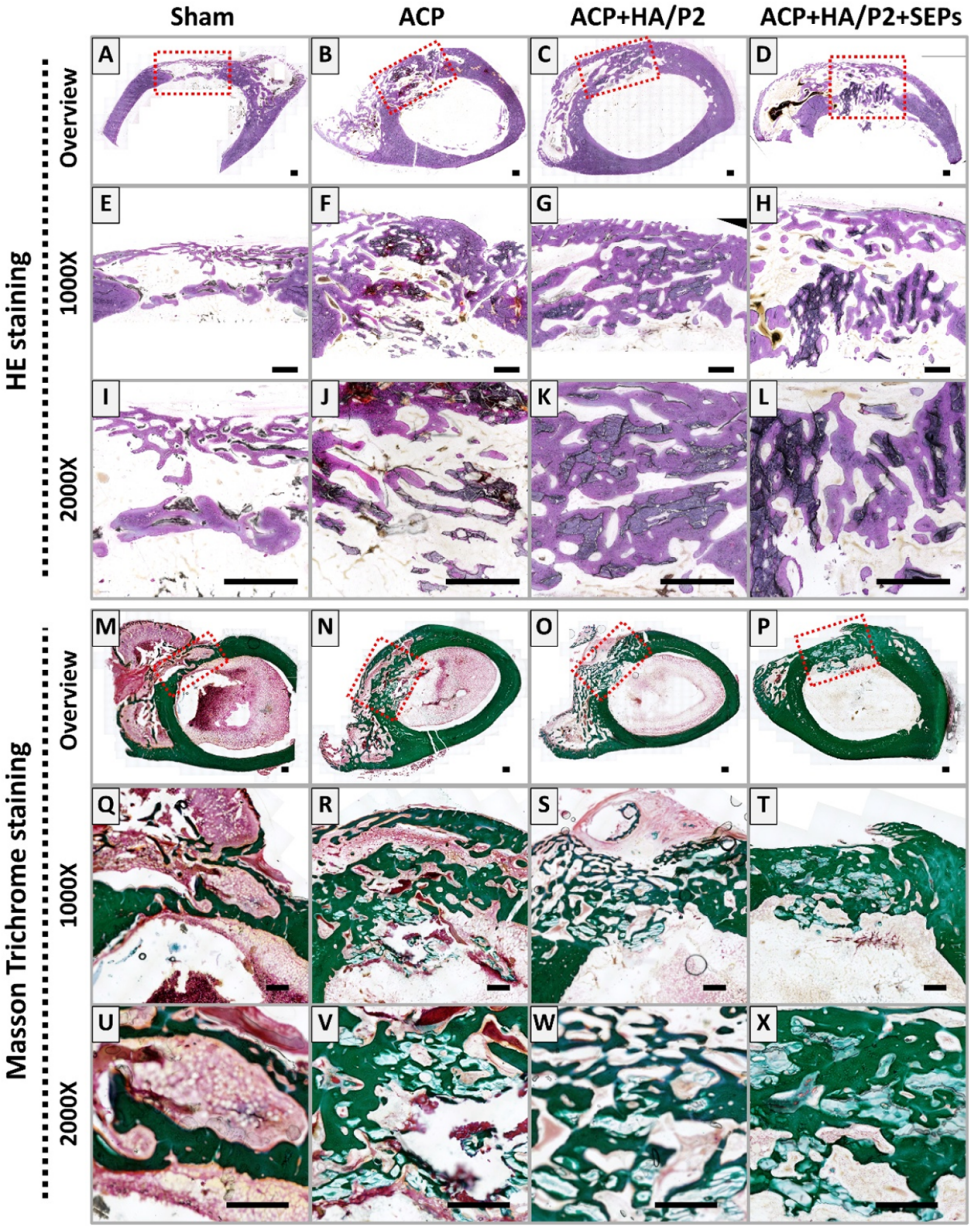
Representative histological staining of bone regeneration in critical-sized rabbit femur defects at 6 weeks postoperation. HE staining (upper panel) and Masson Goldner Trichrome staining (bottom panel) for bone regeneration in defect areas with different magnifications (A–D and M–P, overview; E–H and Q–T, 1000×; I–L and U–X, 2000×). Scale bar: 500 μm.

### 3.3 Bone Regeneration of Critical-Sized Bone Defects in the Calvarial Model

Considering that long bones and lamellar bones have different blood supply sources and bone marrow volumes, a similar critical-sized defect model was established on rabbit calvaria. Micro-CT virtual sectioning, quantitative analysis, and MGT staining in Figures 7 and 8 demonstrated bone regeneration in different groups. Despite a notable difference existing in Tb.Sp between ACP and ACP + HA/P2 groups via quantitative micro-CT analysis (Figure 8E,K), the superior bone regeneration behavior was observed in the ACP + HA/P2 + SEPs group compared to other groups (also identified in Figure 8G and Tables 2 and 3), characterized by a highly integrated bone-material fusion zone (Figure 7G–I) and an ignorable uncalcified gap between grafts and defect margins (Figure 7J–L).

**Figure 7:**
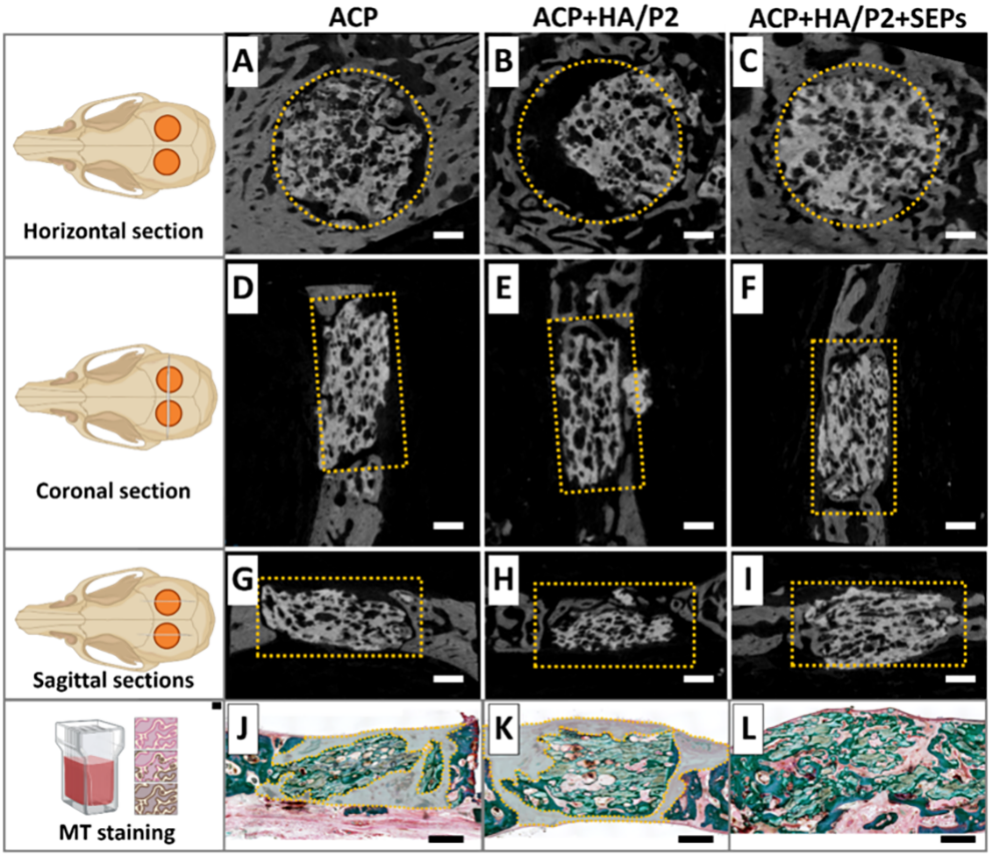
Micro-CT virtual sectioning and histological staining of critical-sized bone defects in rabbit calvariae at 6 weeks postoperation. (A–C) Bone regeneration and mineralization of the horizontal section of calvarial defects. (D–F) Bone regeneration and mineralization of the coronal section of calvarial defects. (G–I) Bone regeneration and mineralization of the sagittal section of calvarial defects. (J–L) Masson Goldner Trichrome staining for bone regeneration in calvarial defects. Orange dotted frame in (A–I): original bone defect area. Orange dotted frame with transparent blue area in (J–L): unmineralized tissue between ACP scaffold and innate bone. Scale bar = 500 μm.

**Figure 8:**
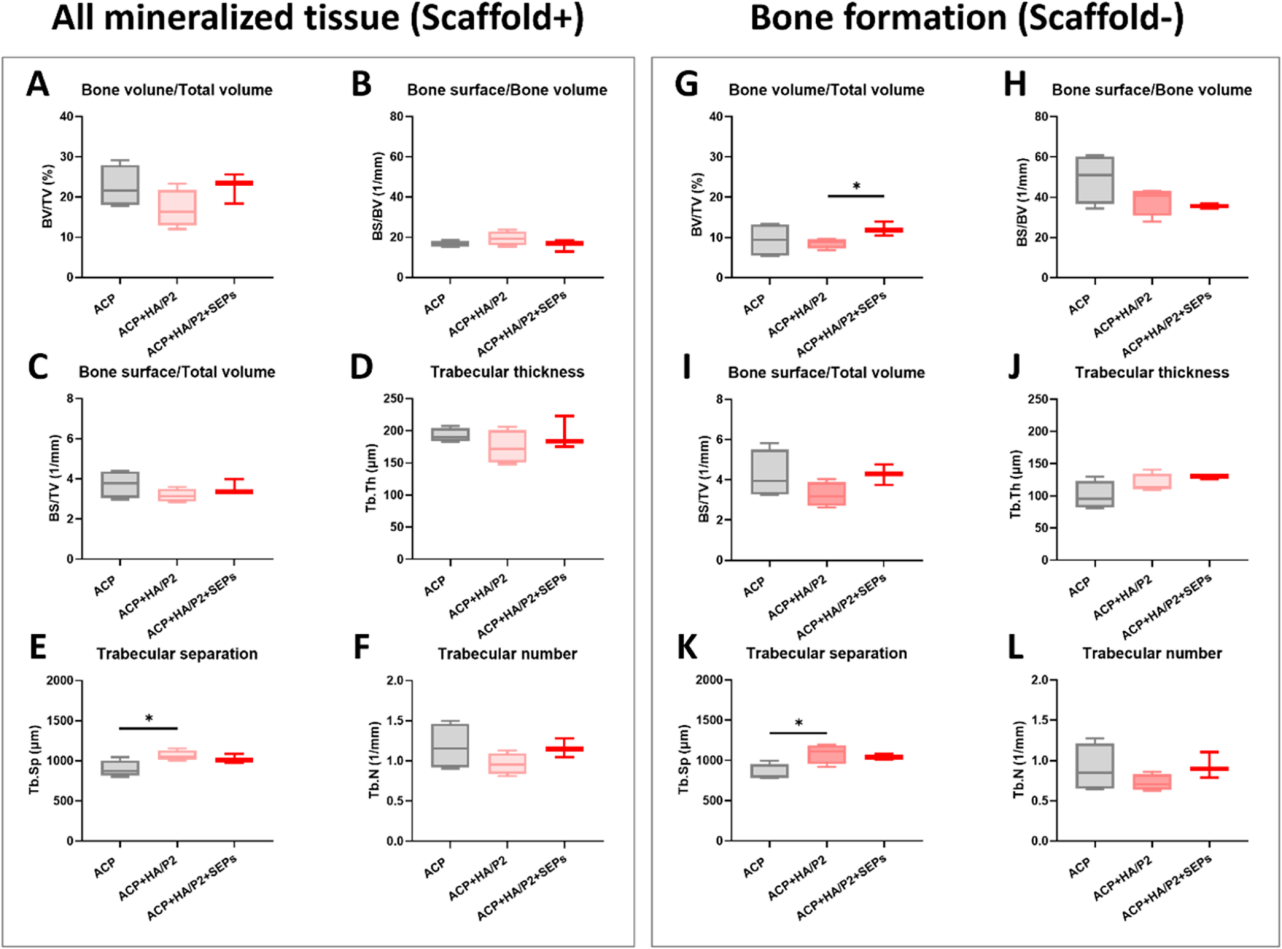
Micro-CT volumetric analysis of bone regeneration in a cylinder region of interest (ROI) with a diameter of 4.0 mm at 6 weeks postoperation in the rabbit calvarial defect model. The bone formation was analyzed with (A and G) Bone volume/Total volume, (B and H) Bone surface/Bone volume, (C and I) Bone surface/Total volume, (D and J) Trabecular thickness, (E and K) Trabecular separation, and (F and L) Trabecular number. All analyses were performed on mineralized tissue with ACP scaffolds (in the left panel) and only on bone tissue without an ACP scaffold (in the right panel). *p < 0.05, **p < 0.01. *N*_ACP_ = 4, *N*_ACP+HA/P2_ = 4, *N*_ACP+HA/P2+SEPs_ = 4.

### 3.4 Bone Regeneration of Critical-Sized Bone Defects in the Femoral Model with Graft Displacement

After sampling, some samples showed mild extracortical calcification. Following micro-CT scanning, some samples were identified as exhibiting graft displacement and were excluded from the analysis in Section 3.2. Although these samples did not reach the experimental expectations, they provided direct evidence for graft-induced osteogenesis inside bone marrow. Micro-CT images of these samples are shown in Figure 9, which clearly demonstrate that the grafts have displaced into the bone marrow. Such displacement did not result in an uncalcified gap between cortical bone and ACP grafts but rather induced bone-graft bridging, particularly in the HA hydrogel-infused ACP groups (Figure 9G,H).

**Figure 9:**
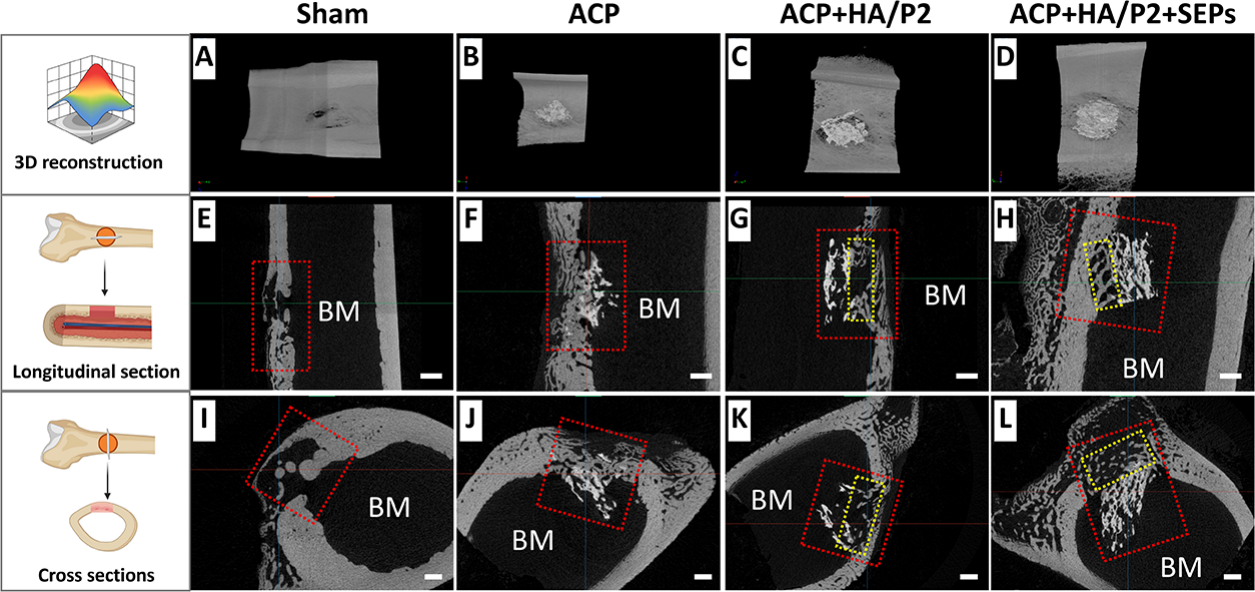
Micro-CT images and virtual sectioning of critical bone defects in rabbit femurs at 6 weeks postoperation with scaffold displacement. (A–D) Reconstruction of bone regeneration in rabbit femur defects. (E–H) Bone regeneration and mineralization of the longitudinal section of femur defects. (I–L) Bone regeneration and mineralization of the cross-section of femur defects. Red dotted frame: original bone defect area; Yellow dotted frame: bone bridge between innate bone and scaffolds. Scale bar = 1.0 mm.

## 4 Discussions

Reducing the environmental impact of graft production is an increasingly important objective in biomaterials development, alongside safety and clinical efficacy.^33,34^ Upcycling eggshells into calcium phosphate scaffolds directly addresses this by diverting a high-volume food byproduct from waste streams and reducing the demand for virgin mineral resources and associated extraction processes.^35^ Additionally, the low-temperature fabrication route employed here eliminates the energy-intensive sintering process commonly used in traditional ceramic manufacturing, thereby reducing energy consumption and emissions.^36^ By combining waste-to-resource utilization with reduced energy input, this approach offers a more sustainable pathway for generating clinically relevant bone substitutes than conventional, de novo-synthesized, or high-temperature-processed ceramics.^37^ While this study provides robust evidence of the technical feasibility of eggshell upcycling, transitioning this laboratory-scale demonstrator into a definitive clinical solution will require addressing several real-world translational hurdles.

In our previous work, ACP-derived particles were synthesized from hydroxyapatite (HAp) and eggshell waste and subjected to biological evaluation.^14^ These *in vitro* and *in vivo* (rat calvarial model, *submitted*) studies demonstrated that eggshell ACP particles enriched with Mg and Sr elements exhibited superior osteoinductivity compared to HAp-derived ACP particles. Accordingly, eggshell ACP materials were used in the following studies. Notably, the eggshell ACP particle agglomerates were small in size (average diameter ∼ 13 μm), which may limit clinical handling, spatial support for bone regeneration, and the establishment of functional vascularization.^14,38^ Although compressing small ACP particles into larger granules (Φ0.5–1.0 mm) is theoretically feasible, industrial-scale production would require higher energy input and specialized molds. Constructing porous ACP scaffolds, therefore, offers a more practical translational strategy. By adjusting the spatial holder’s proportion, scaffolds with different internal porosities can be obtained. However, due to the heat- and moisture-sensitive nature of ACP materials, removing conventional space holders thermally or aqueously will result in the loss of the amorphous phase. To address this issue, ice microspheres were introduced as space holders, and porous ACP scaffolds were fabricated via pressure densification at subzero conditions. This strategy avoided the introduction of harmful chemical reagents and minimized moisture-related ACP recrystallization, thereby preserving the amorphous characteristics (Figure 3).^14^ To determine the applicable porosity, we prepared scaffolds with porosities ranging from 40% to 80%. Micro-CT showed that pores were largely unconnected below 50% porosity, whereas scaffolds became highly fragile above 70% porosity (data not shown; available on request). Based on these findings, a porosity of 60% was used in the in vivo study. Notably, the porosity indicators showed that the porous scaffolds prepared by using the ice microsphere template method in this study have highly similar internal structures across batches, providing an experimental basis for the industrial-scale manufacturing of homogeneous products.

Combining inorganic bone grafts with hydrogel can further promote bone regeneration compared with using inorganic grafts alone.^39^ Given the rich marrow and blood supply in rabbit femurs, HA hydrogel was selected as an ideal biomolecule (SEPs+P2) carrier for ACP scaffolds. HA can bind fibrins to stabilize blood clots, enhance the homing, attachment, and proliferation of bMSCs, and promote mineralized tissue repair via the HA-CD44 signaling axis.^40–43^ In addition, the synthetic proline-rich peptide P2, which exhibits intrinsically disordered protein (IDP) features, has shown stronger osteoinductive activity than animal-derived enamel matrix derivative (EMD; Emdogain, Institute Straumann AG, Switzerland). Using a synthetically produced peptide rather than one derived from porcine tissue avoids batch-to-batch biological variability and animal-borne contaminants, improves compositional control and regulatory traceability, and circumvents ethical or religious constraints associated with animal-derived products.^19^ Moreover, SEPs extracted from eggshell membrane enhanced osteoblast proliferation and ECM synthesis, positioning eggshell-derived ACP and SEPs as a single-origin and complementary biomaterial package.^44^ Therefore, following our previous approach, a partially cross-linked HA hydrogel loaded with P2 and SEPs was prepared further to enhance the bone-repair capacity of the composite grafts.^31,45^

In the femoral defect model, the abundant blood supply resulted in rapid bleeding upon marrow exposure, which submerged the grafts and potentially facilitated new bone formation in the presence of ACP scaffolds. Even without ACP scaffolds, bone-marrow-derived blood can promote extracortical calcification (Figures 4 and 5). Interestingly, ACP scaffolds combined with HA/P2 did not exhibit superior pro-osteogenic effects compared with ACP scaffolds alone. However, adding SEPs to the graft system clearly improved bone formation, as evidenced by a reduction in the level of trabecular separation (Tb.Sp) (Figure 5E,K). While micro-CT provided an overall quantitative assessment of osteogenesis (regardless of inclusion of scaffold signal), histological evaluation of new bone, although inherently selective, remains essential. In this study, histology focused on bone–material interfaces. Consistent with prior reports, HA/P2 facilitated deposition and mineralization of collagenous tissue (Figure 6K,W).^46–48^ Furthermore, more mature bone and intact bone-graft fusion zones were observed in the ACP + HA/P2 + SEPs group (Figure 6L,X), underscoring the in vivo osteogenic potential of SEPs, which aligns with our previous in vitro findings.^17^ This superior integration is fundamentally linked to the concept of biomaterial confinement, a critical prerequisite for GBR.^18^ The HA/P2/SEPs hydrogel matrix functions as a physical seal, providing essential spatial stability within the defects and within the interior of ACP scaffolds. As demonstrated by the complete encapsulation of ACP scaffolds in our histological sections, this confinement effectively restricted fibrous tissue infiltration while maintaining a high local concentration of bioactive P2 and SEPs. Such a localized microenvironment transformed the tissue-scaffold interface into a highly inductive niche, thereby facilitating the observed robust bone-graft fusion. To further contextualize these findings within the clinical landscape, it is essential to compare our system with established modalities such as autologous platelet-rich plasma (PRP) or platelet-rich fibrin (PRF) and recombinant growth factors. While autologous PRP/PRF systems are clinically accessible and rich in growth factors like PDGFs and VEGFs, they are characterized by significant batch-to-batch variability and unpredictable degradation profiles, which can compromise the consistency of bone healing.^49,50^ Our partially cross-linked HA/P2 hydrogel addresses these deficiencies by offering superior compositional control and tunable mechanical stability. Besides, although potent osteoinductive agents like Bone Morphogenetic Proteins (BMPs) and bMSCs are highly effective, their clinical use is often hindered by high costs, complex regulatory pathways, and significant safety risks, most notably the potential for ectopic bone formation and excessive inflammatory responses associated with high-dose BMP-2 application and the donor-to-donor heterogeneity of bMSCs.^51–53^ By leveraging the synergistic, mild osteoinductive effects of eggshell-derived ACP and SEPs, our platform provides a safer and more sustainable alternative that avoids the risks of ectopic ossification while maintaining robust regenerative potential.

Bone regeneration depends not only on the graft composition but also on the local tissue microenvironment. Bone healing dynamics are inherently site-specific, governed by distinct embryonic ossification pathways: the femur predominantly undergoes endochondral ossification under physiological loading, whereas the calvarium relies on intramembranous ossification in a nonload-bearing environment.^54,55^ The choice of a 5.0 mm diameter for both models in this study was justified by the published literature^56–59^ and our preliminary pilot data, which demonstrated that a 5.0 mm circular defect in the rabbit calvarium failed to achieve functional bony bridging within 9 months of healing (data not shown). This is consistent with established literature defining the critical-size threshold for rabbit cranial defects to ensure that observed regeneration is graft-induced rather than spontaneous.^21,60^ While the absence of a calvarial Sham group, necessitated by anatomical space constraints, precludes an absolute baseline comparison at this site, our interpretation focuses on the relative regenerative efficacy among scaffold formulations. This approach avoids potential bias introduced by comparing calvarial experimental groups with femoral Sham parameters, thereby respecting the fundamental biological differences between intramembrane and endochondral repair.

Compared with femoral defects, calvarial defects showed pronounced gaps between grafts and defect margins in the ACP and ACP + HA/P2 groups, indicating incomplete bone–graft fusion (Figure 7). With a reduced blood supply, the partially cross-linked, granulated HA hydrogel may disrupt blood clot formation and the homing of osteogenic lineage cells. The disruption of blood supply caused by defect preparation also creates a hypoxic microenvironment, which can inhibit the metabolic transformation of bMSCs and impair subsequent bone repair (Figure 8E,K).^61,62^ Cross-linking and granulation of HA were intended to increase stiffness and resistance to enzymatic degradation, thereby helping establish and maintain a favorable osteogenic microenvironment.^31,63^ Tuning the degree of cross-linking and the HA granule size may be a practical strategy to enhance early repair at sites with limited perfusion. Nevertheless, SEPs consistently improved bone–graft interactions and yielded more mature fusion zones than other groups, likely via enhanced chemotaxis, proliferation, and ECM synthesis of osteogenic lineage cells mediated by the complex active components in SEPs.^17^ These effects appear to convert the cortical margin from a primarily osteoconductive boundary into a more inductive niche, enabling new bone formation that extends into scaffold pores and improves bone–scaffold continuity. Notably, the pro-osteogenic effect of SEPs was evident even under suboptimal conditions with partial graft displacement, where SEPs promoted bone bridging between cortical bone and ACP scaffolds (Figure 9H), further supporting an inductive contribution at the bone-graft interface. In our preliminary *in vivo experiments, the Sham group did not exhibit notable spontaneous osteogenic behavior after 6 weeks of healing (data not shown;* available upon request). Because of the limited area of the rabbit calvarium, the Sham group was excluded from the calvarial model. Instead, we focused on comparing the differences in osteogenesis across ACP scaffold-containing groups.

From a translational perspective, investigating the activity of individual proteins within SEPs is inefficient and costly. Our prior work showed that eggshell membrane proteins derived from different feeding conditions (organic vs industrial) have largely identical protein profiles and remarkably similar pro-osteogenic functions,^17^ suggesting that eggshells from diverse sources can be treated as equivalent raw materials. The high-value-added conversion of both inorganic and organic components in eggshell waste—combined with appropriate flexible biomaterials—can thus yield practical biomaterial packages for sustainable bone regeneration.^64,65^ This waste-to-value approach reduces the bioburden of bone regeneration therapies. It circumvents religious and ethical concerns, offering an environmentally friendly and promising pathway for the development of novel bone-graft materials.

## 5 Limitations and Future Directions

The rabbit femoral defect model provides a mechanically active context that enables evaluation of graft osteogenic capacity under physiological cyclic loading. However, it is important to acknowledge that the monocortical critical-sized defect used in this study represents a load-sharing rather than a full-load-bearing environment. The remaining intact cortical bone and the surrounding soft tissue provide significant intrinsic stability, preventing the graft from experiencing the extreme biomechanical demands typically associated with complete segmental defects.^66–68^ Consequently, while the eggshell-derived ACP scaffolds demonstrated robust mineralized tissue formation and integration, claims regarding their ultimate load-bearing ability in the context of major limb reconstruction should be interpreted with caution. In true segmental models, which involve a circumferential loss of bone, scaffolds must possess higher inherent mechanical strength and are often paired with rigid internal or external fixation to withstand full axial and torsional forces.^69^ In this study, the high propulsive forces generated during ambulation led to postoperative femoral fractures in some animals. The relative paucity of cancellous bone within the femoral medullary canal predisposes it to graft displacement, compromising subsequent osteogenic assessments and raising animal-welfare concerns due to potentially avoidable euthanasia. Additionally, as noted during the discussion of site-specific healing, a limitation of this study is the absence of a concurrent Sham control group in the calvarial model. Although our preliminary studies confirmed negligible spontaneous healing in 5.0 mm cranial defects at 6 weeks, the absence of a Sham control in the main experiment necessitated a more cautious interpretation of the absolute regenerative rate. The different ossification characteristics of the cranium and femur mean that site-specific controls are ideal for eliminating potential interpretive bias. Future studies using larger animal models or modified defect arrays will be required to incorporate these controls and further validate the findings in both load-bearing and nonload-bearing contexts. In parallel, we will develop personalized, sustainability-focused graft strategies by tuning the parameters of the flexible SEP-containing hydrogel, in combination with the eggshell-derived ACP scaffold, to align with defect location, size, blood supply, and loading environment. To translate this proof of concept into a standardized clinical solution, future efforts must address the logistical and regulatory complexities of waste-to-resource pathways. First, establishing a standardized collection and pretreatment protocol for food waste is essential to ensure the chemical consistency and biological safety of eggshell-derived precursors across different batches and geographic sources. Second, although our low-temperature fabrication preserves the bioactive amorphous phase, the scalability of subzero-pressure densification remains to be optimized for industrial-scale production. Comprehensive life-cycle assessments (LCAs) and cost-benefit analyses compared with traditional synthetic grafts will be necessary to validate the long-term economic and environmental viability of these materials. Finally, navigating the regulatory landscape for ’upcycled’ biomaterials will require rigorous validation of viral and prion deactivation during the conversion process to meet the stringent safety standards for clinical implantation. Addressing these interdisciplinary challenges will be the key to transforming this sustainable platform into a definitive alternative for global bone repair.

## 6 Conclusions

In rabbit femoral and calvarial critical-size defect models, eggshell-derived ACP scaffolds supported substantial bone regeneration. Pairing these scaffolds with a P2 peptide-functionalized hyaluronic acid hydrogel enriched with eggshell membrane proteins (SEPs) was found to enhance early bone formation and promote the effective encapsulation of the scaffolds within mineralized bone tissue. By upcycling the inorganic and organic fractions of eggshell waste, this study delivered a sustainable, single-origin biomaterials platform that, upon addressing logistical and regulatory challenges, offers a definitive solution for early bone regeneration.

## Data Availability

The raw data required to reproduce these findings cannot be shared at this time, as they also form part of an ongoing study.
